# ApoNecV: A macro for cell death type differentiation

**DOI:** 10.1111/jmi.13386

**Published:** 2025-01-24

**Authors:** Marketa Kolarikova, Barbora Hosikova, Jiri Tesarik, Katerina Langova, Hana Kolarova

**Affiliations:** ^1^ Department of Medical Biophysics Faculty of Medicine and Dentistry Palacky University Olomouc Czech Republic

**Keywords:** apoptosis, cell death, microscopy, necrosis

## Abstract

The evaluation of large experimental datasets is a fundamental aspect of research in every scientific field. Streamlining this process can improve the reliability of results while making data analysis more efficient and faster to execute. In biomedical research it is often very important to determine the type of cell death after various treatments. Thus, differentiating between viable, apoptotic, and necrotic cells provide critical insights into the treatment efficacy, a key aspect in the field of drug development. Fluorescent microscopy is perceived as a widely used technique for cell metabolism assessment and can therefore be used to investigate treatment outcomes after staining samples with cell death detection kit. However, accurate evaluation of therapeutic results requires quantitative analysis, often necessitating extensive postprocessing of imaging data. In this study, we introduce a complementary tool designed as a macro for the Fiji platform, enabling the automated postprocessing of fluorescent microscopy images to accurately distinguish and quantify viable, apoptotic, and necrotic cells.

## 1 INTRODUCTION

The determination of the proportion of viable versus non‐viable (apoptotic and necrotic) cells in cell samples can help to evaluate the treatment outcome, especially in cancer therapies where inducing tumour cell death is a primary goal. Importantly, the distinction between apoptosis known as programmed cell death, and necrosis known for uncontrolled cell death, provides insights into its mechanism, which is vital for drug development and prediction of potential side effects. Unlike apoptosis, necrosis can trigger inflammatory responses which underlines the importance of cell death type identification that influences drug concentration and treatment decisions. Apart from cancer treatments, distinguishing between apoptosis and necrosis helps to understand disease progression as well as impact of therapeutics in neurodegenerative disorders or pathogen‐induced cell death.^1^, ^2^

The type of cell death after different treatments plays a crucial role in evaluation of the treatment efficacy. Depending on conditions, cells can undergo apoptotic or necrotic death with different outcomes for the patient. There are several ways to differentiate between apoptotic and necrotic cells. The method employed by Annexin V‐CY3^TM^ Apoptosis Detection Kit (APOAC, Sigma Aldrich) involves the use of two labels: Annexin‐Cy3.18 (AnnCy3) and 6‐Carboxyfluorescein diacetate (6‐CFDA). The combination of these dyes allows for the distinction between live, apoptotic, and necrotic cells based on their fluorescence patterns. The Annexins are a group of homologous proteins that bind phospholipids in the presence of calcium. During apoptosis (programmed cell death), certain annexins, like annexin V, translocate to the outer leaflet of the plasma membrane where they bind to exposed phosphatidylserine. This process is a key marker of early apoptosis.^3^

AnnCy3 has a high affinity for phosphatidylserine, which is externalised to the outer leaflet in early apoptotic cells, and to inner and outer membrane leaflets of necrotic cells. The other probe, 6‐CFDA, is used to measure viable cells as it enters living cells in its non‐fluorescent form, which is then hydrolysed by present esterases to green fluorescent 6‐CF (cole). Early apoptotic cells also retain membrane integrity, allowing 6‐CFDA to enter and provide green signal upon conversion to 6‐CF, but they will also show red fluorescence due to AnnCy3 binding to exposed phosphatidylserine. In necrotic cells, the loss of membrane integrity results in the leakage of intracellular content leading to a loss of green fluorescence, but providing red fluorescence induced by AnnCy3 binding.^3^, ^4^ Thus, live cells are characterised by green fluorescence (6‐CF), while necrotic cells provide red fluorescence (AnnCy3). Cells undergoing apoptosis render both red (AnnCy3) and green (6‐CF) signal.^4^

Although this APOAC kit offers an advantage of simplicity and easy step‐by‐step protocols, the data collection and evaluation can be robust. To correctly assess the treatment outcomes, large datasets are usually needed where hundreds to thousands of cells per sample need to be evaluated after being imaged with a confocal microscope. Therefore, a simple and fast method with reliable results would be useful to replace the outdated method of manual counting individual cells in green, red or merged channel to find out the resulting treatment output. Here, an ApoNecV macro is presented to process microscopy images with subsequent cell death analysis based on fluorescent signal.

## 2 METHODS AND MATERIALS

### 2.1 Cell culture

The HeLa cell line (CCL‐2, ATCC, USA), derived from cervical cancer cells, was used in the 10th batch. Cells were grown on a high performance #1.5 cover glass bottom 24‐well plate (Cellvis #P24‐1.5H‐N) specifically designed for confocal microscopy. Into each well, 50,000 cells were seeded to adhere for 24 h. Cells were cultivated in high‐glucose DMEM (Dulbecco's Modified Eagle's Medium, D7777, Sigma‐Aldrich) medium supplemented with 10% FBS (Fetal Bovine Serum, F7524, Sigma‐Aldrich) and 1% Penicillin‐Streptomycin (P4333, Sigma‐Aldrich) in the incubator tempered to 37°C and 5% CO_2_.

### 2.2 Photodynamic treatment

To induce necrotic cell death, ZnPc (2, 3, 9, 10, 16, 17, 23, 24‐Octakis [(2‐(triethylammonio)ethyl)sulfanyl] phthalocyaninato] zinc(II) Octaiodide), synthesised by A. Cidlina,^5^ was administered in 5 µM concentration and incubated with cell samples for 1 h in the incubator tempered to 37°C and 5% CO_2_. After 1 h of incubation, cells underwent 5 min of irradiation with 21 mW/cm^2^ using red LED light (660 nm, patent number CZ302829B6) to activate ZnPc, which then created reactive oxygen species leading to cell death.^6^ At the same time, unaffected negative control cells were imaged under the same conditions providing control sample data.

### 2.3 Staining

According to the protocol, cell staining procedure was performed at room temperature for 15 min without light exposure.^4^ Control and treated cell samples were incubated with both probes simultaneously and observed with a fluorescent microscope immediately after incubation time ended.

### 2.4 Imaging

Cells were imaged in Phosphate Buffer Saline (PBS) using a confocal spinning disk microscope Yokogawa CSU‐X1 (ZEISS Zen Blue software) with an objective EC Plan‐Neofluar 10× (0.3 NA). 6‐CF (ex 495nm/em 520 nm) was excited with 488 nm laser, while AnnCy3 (ex 550 nm/em 570 nm) was excited with 561 nm laser. The light source intensity was set for both channels to 50% with gain set to the value of 750. Per each sample, 3 images were acquired with approximately 500 to 1000 cells to be analysed.

To accelerate and streamline the cell death analysis, ApoNecV macro was created and applied using Fiji platform. To compare the results and confirm the reliability of this macro, the same set of images was evaluated manually on individual cell counting principle. To ensure unbiased results, the manual and digital processing of the images was performed by two individuals.

### 2.5 ApoNecV: a platform for cell death type detection

ApoNecV is a Fiji^7^ macro that enables assessment of viable, apoptotic and necrotic cells. To obtain correct data, cell samples must be stained with APOAC kit (AnnCy3 and 6‐CFDA) according to the official protocol. Also, to obtain precise results using ApoNecV, the images must be acquired with a 10× objective (0.3 NA). 40×, 60× and 100× objectives are not verified for ApoNecV functionality and could therefore generate inaccurate processing.

The filters must be set accordingly to fluorescence features of the probes: 6‐CF (ex 495nm/em 520 nm), excited with 488 nm laser and AnnCy3 (ex 550 nm/em 570 nm) excited with 561 nm laser. The light source intensity and gain should be optimised based on each experiment's conditions. Per each sample, several images should be acquired to ensure reproducibility and correct results.

Prior to imaging of samples, single label controls should be set to make sure that no bleedthrough occurs due to incorrect settings of filters. Special attention should be given to the issue of autofluorescence, which should be subtracted using correct microscope settings. Images acquired under these conditions can further proceed for ApoNecV operation.

## 3 RESULTS

### 3.1 Background subtraction and deconvolution

The first processing in ApoNecV macro employs background subtraction and deconvolution algorithms. Background from both channels was subtracted using Rolling Ball Radius algorithm^8^ set to values of 50 for 6‐CF and 30 for AnnCy3. Once background was subtracted, deconvolution could proceed. If the setting of microscope is set correspondingly to the settings described above in section Imaging, the Rayleigh resolution of input images (must be a stack image of green, red and transmitted light channel) will be identical and there is no need to further edit data to generate point spread function (PSF), which is needed to deconvolve input images. PSF characterises the respond of an imaging system to a point source of point object. It describes the light distribution from a single point in the object space as it appears in the image space representing system's spatial resolution and thus ability to reproduce fine details. Accurate PSF modelling is crucial as it enable the correction of image distortions caused by optical aberrations and other imperfections of imaging systems.^9^

**FIGURE 1:**
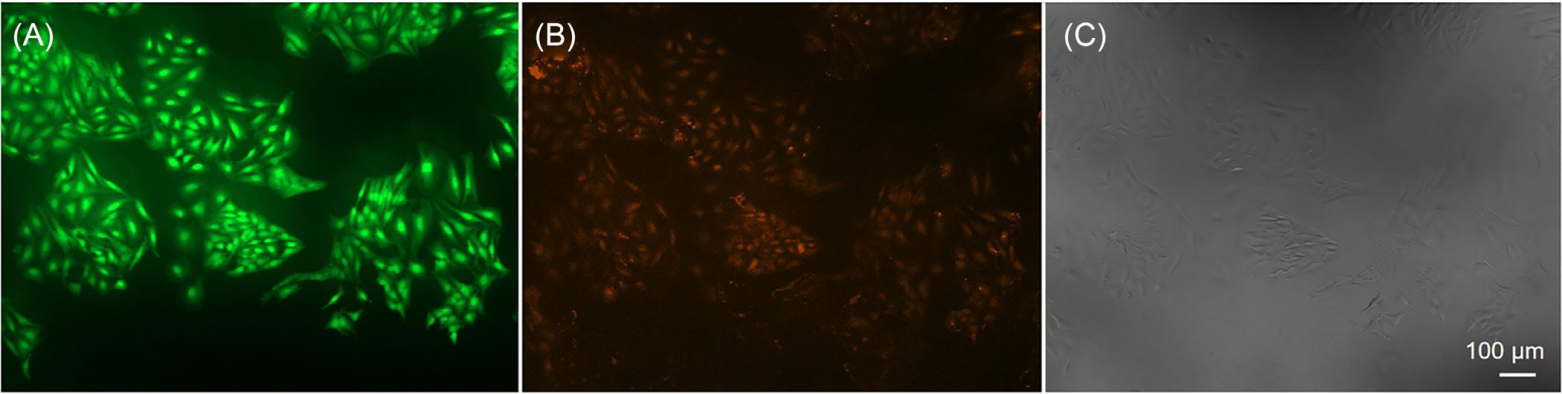
Raw images of HeLa cells stained with APOAC kit. Channel A – green fluorescence of 6‐CF. Channel B – red fluorescence generated by AnnCy3 dye. Channel C – transmitted light channel. Images were acquired at 100× magnification.

**FIGURE 2:**
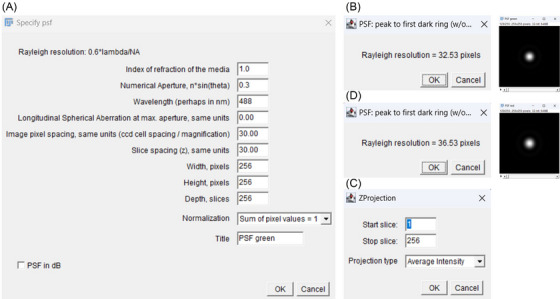
Process of PSFs generation. Data for Diffraction PSF 3D plugin (A), generation of Rayleigh resolution (B, D) and Z‐projection of PSF (C).

Figure 1 shows raw images of HeLa cells for 6‐CF (green channel), AnnCy3 (red channel) and transmitted light channel. Both AnnCy3 and 6‐CF images proceeded to deconvolution, as described in Figure 2. In order to deconvolve images, correct PSFs had to be first generated for both channels. All these steps are automated in ApoNecV upon opening a stack image of green, red and transmitted light channels.

Using Diffraction PSF 3D plugin,^10^ data such as Index of refraction of the media (1.0 for air), Numerical Aperture (0.3 using 10× objective Plan Neofluar), Wavelength (488 nm laser was used to excite 6‐CF) and Title (PSF green) were filled in for 6‐CF (green) channel (Figure 2A) to generate Rayleigh resolution (32.53 pixels) (Figure 2B). After accepting Rayleigh resolution value, a 32‐bit 64 MB image titled PSF green was generated, which was used to generate Z‐projection set to Average Intensity to generate PSF for green channel (Figure 2C). For red channel AnnCy3, the same dataset was used except for Wavelength, as 561 nm laser was used to excite AnnCy3 and Title, which was named PSF red instead. The resulting value of Rayleigh resolution was 36.53 pixels (Figure 2D).

**FIGURE 3:**
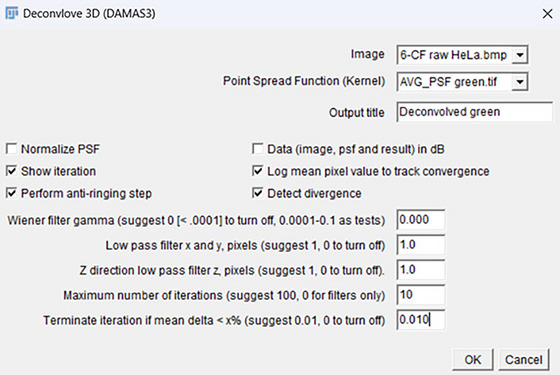
Using iterative deconvolve 3D, a window pops up with the following information to fill in.

**FIGURE 4:**
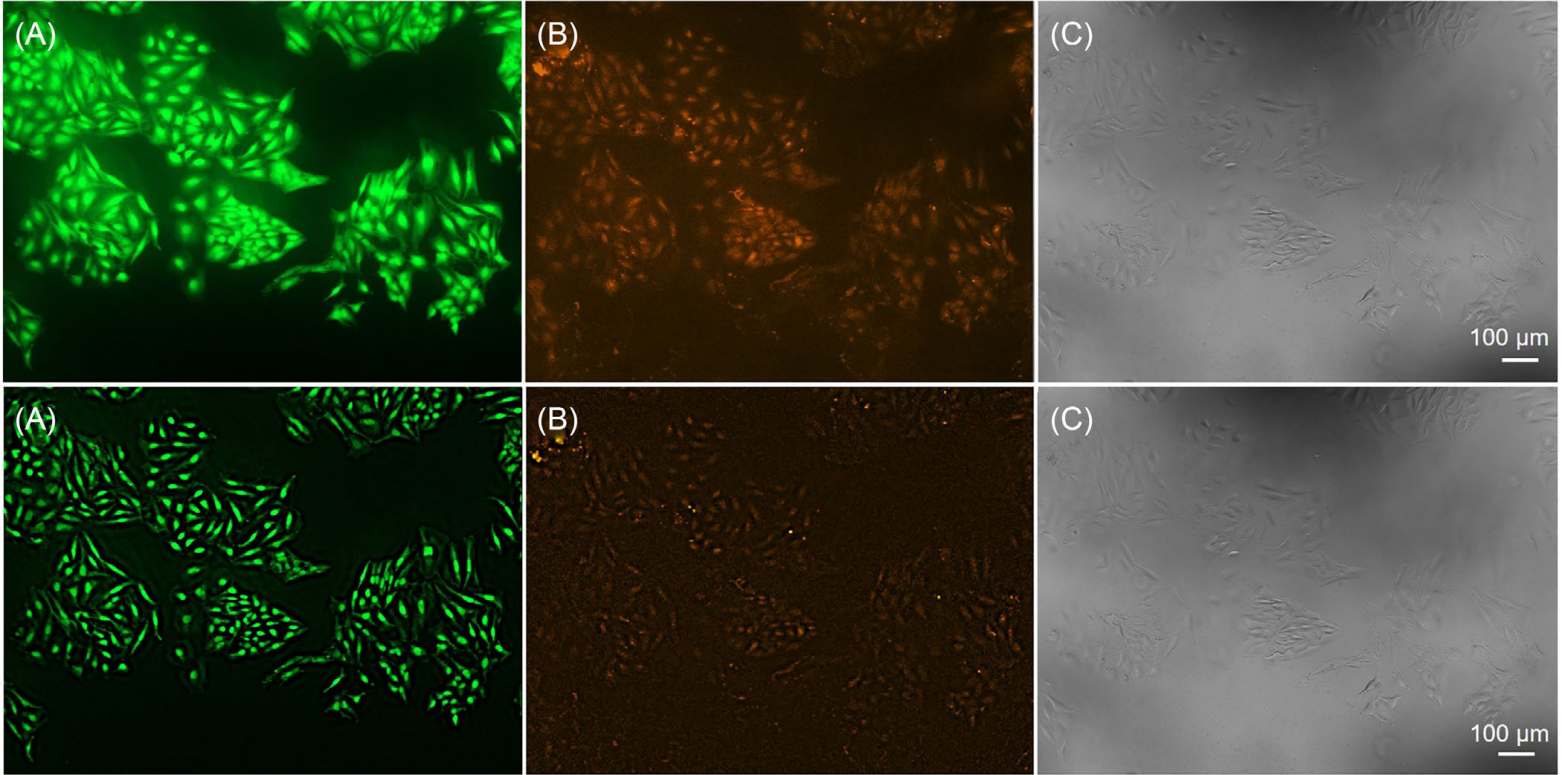
A comparison of raw (top line) and deconvolved (bottom line) images of HeLa control cells stained with APOAC kit. Channel A – green fluorescence of 6‐CF. Channel B – red fluorescence generated by AnnCy3 staining. Channel C – transmitted light channel. Images were acquired at 100× magnification.

Once PSFs were generated, images proceeded for deconvolution using Iterative Deconvolve 3D plugin (ImageJ, 2005)^11^ operation. In this step, Image and Point Spread Function must match to deconvolve correctly. Also, Wiener filter gamma was set automatically to 0.000, Low pass filter x and y to 1.0, *Z* direction low pass filter z to 1.0, Maximum number of iterations was set to value of 10, and termination of iterations was 0.010, as shown in Figure 3. Once all these data were filled in, the deconvolved image was generated (Figure 4).

### 3.2 Mask generation

**FIGURE 5:**
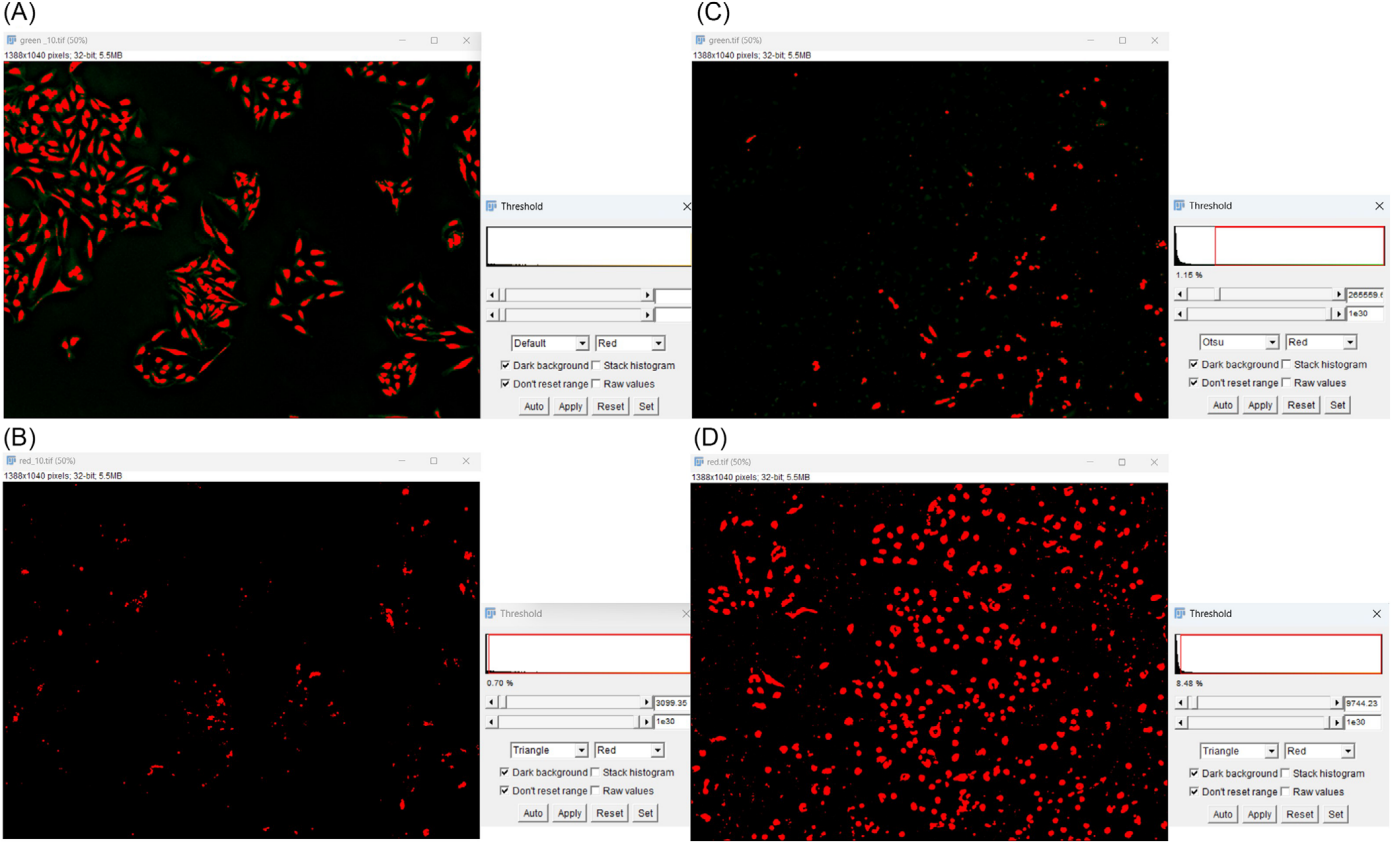
Thresholding algorithms for control (A, B) and treated (C, D) cell samples. Here, Default algorithm was chosen for 6‐CF channel in control sample (A), while for treated sample, an Otsu algorithm was found to be adequate for 6‐CF channel (C). Triangle algorithm was then chosen for both control and treated AnnCy3 channel samples. Images were acquired at 100× magnification.

Once deconvolution was performed, images were smoothed twice, and thresholded with Default (applicable for control cells, 6‐CF channel), Triangle (applicable for control and treated cells, AnnCy3 channel) or Otsu (applicable for treated cells, 6‐CF channel) algorithms, which were found to provide most reliable results as shown in Figure 5. Due to distinct morphological features of viable and apoptotic/necrotic cells together with fluorescent features of both used dyes, a Default thresholding algorithm could not be applied to all the scenarios. Thus, from all the available FiJi thresholding algorithms, Triangle and Otsu were found to fit perfectly for the situations described above. After image thresholding, a conversion to binary masks was performed. A watershed function was then used to separate cells in 6‐CF binary image (control and treated cells) and AnnCy3 binary image (control cells). Importantly, watershed function was not needed in AnnCy3 binary image (treated cells), as the signal from red fluorescence accumulated in smaller formations inside cells and did not optically fuse cells.^7^

### 3.3 Image calculator and particle analysis

**FIGURE 6:**
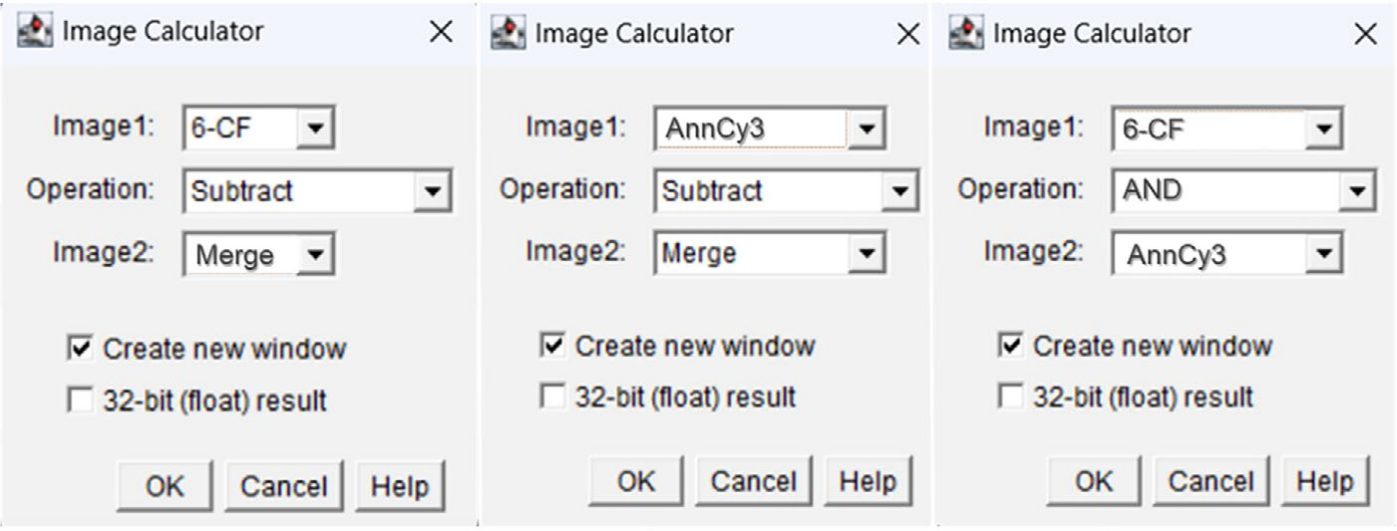
Image Calculator operations with ‘AND’ and ‘Subtract’ functions to generate 6‐CF only, AnnCy3 only and Merge binary images.

**FIGURE 7:**
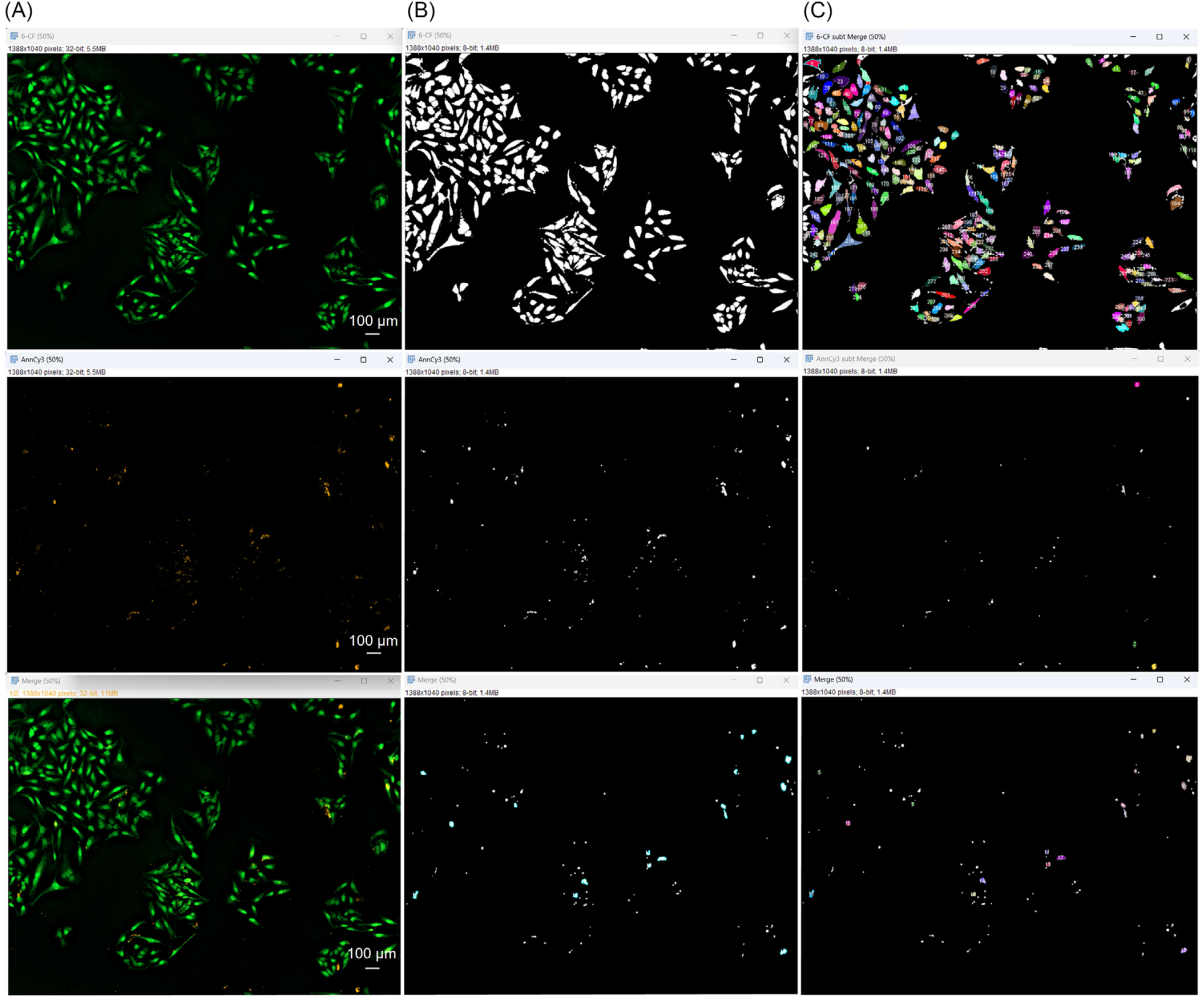
Deconvolved (A), binary (B) and masked (C) images of CF‐6, AnnCy3 and merge channels. Colour with corresponding numbers in overlay images represent cells (viable/apoptotic or necrotic) that were summarised to give a result. Images were acquired at 100× magnification.

Such processed 6‐CF and AnnCy3 binary images were first used to create a Merge image using Image Calculator operation (6‐CF ‘AND’ AnnCy3). This binary Merge image was then processed with a dilate function (1×) and subsequently subtracted from 6‐CF binary image using Image Calculator (6‐CF ‘Subtract’ Merge) to gather binary image with viable cells only.^7^ The operations used within Image Calculator are shown below in Figure 6. Using a function Analyse Particles, size and circularity values were varying from 50–infinity to 100–infinity (size) and 0.10–1.00 to 0.60–1.00 (circularity), as shown in Table 1. The correct fitting of these values assured that low or unspecific signal from the background was not included in particle counting with potentially false results. In the option Show, Overlay Masks were chosen for a better knowledge of what cells were included in analysis. Those that were included are coloured with a corresponding number, while those not included are not numbered and are shown in white (Figure 7C). Here, an appropriate value set for circularity is particularly important to exclude cells left with tiny fragments after subtraction of Merge image (apoptotic) from 6‐CF image (viable). Also, options Display results, Summarise, Exclude on Edges and Include holes must be chosen for a correct analysis.

**TABLE 1:** Circularity and size values for Merge, AnnCy3 and 6‐CF images. The set of circularity values can greatly vary upon the cell treatment due to changes in cell morphology induced by cell death. The correct values are important to obtain meaningful results and may cause a misinterpretation if set incorrectly. These values were used for images obtained with 10× air objective. If used with other settings, results may be falsely affected.

	Negative control	Treatment
Merge	0.2–1; 100–infinity	0.1–1; 50–infinity
AnnCy3	0.3–1; 100–infinity	0.3–1; 100–infinity
6‐CF	0.2–1; 100–infinity	0.6–1; 100–infinity

**FIGURE 8:**
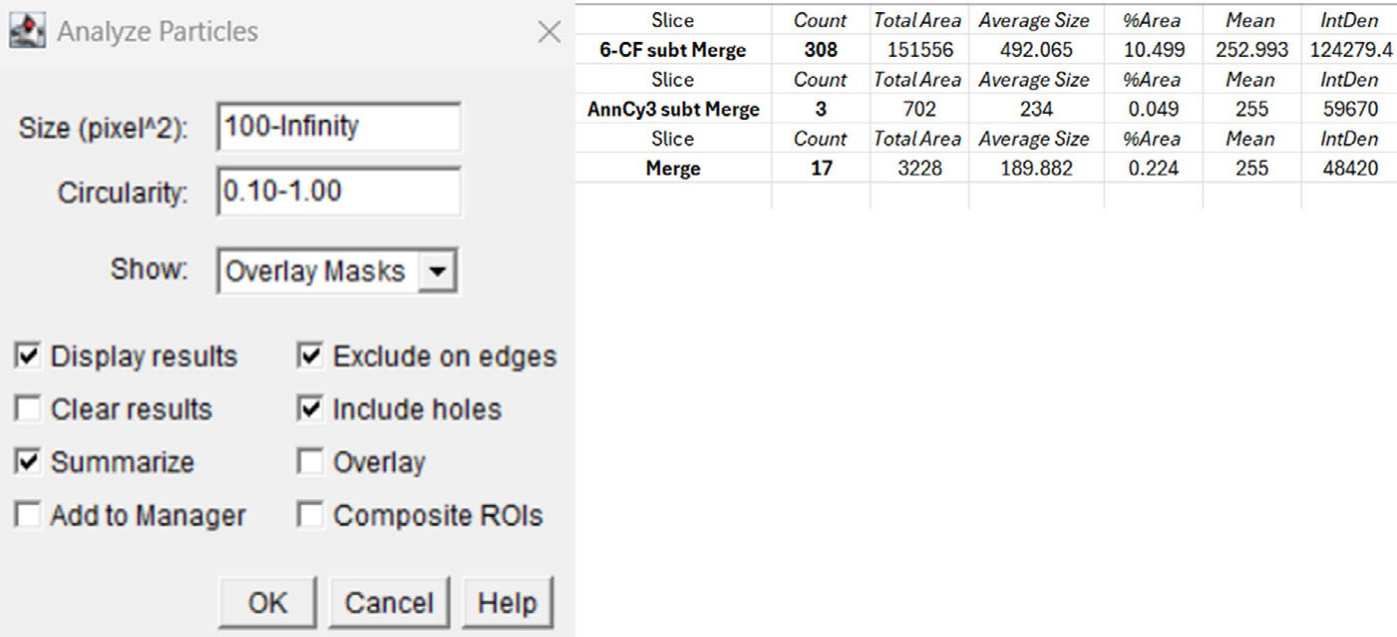
Analyse Particles algorithm with key parameters such as size and cirlularity set on left. The Overlay Masks function was used to generate images with numbers of cells that were included in count analysis. The final outcome of ApoNecV processing can be seen on the right. The total number of viable cells represented by ‘6‐CF subt (subtraction) Merge’ was 308. Necrotic cells (‘AnnCy3 subt Merge’) were found to be 3 and 17 cells were apoptotic (‘Merge’).

To gather information about number of cells undergoing necrosis, the same Merge image was subtracted from AnnCy3 binary image with Image Calculator operation (AnnCy3 ‘Subtract’ Merge), gaining only cells with red signal specific for necrotic hallmarks. Importantly, values for circularity set for AnnCy3, 6‐CF and Merge images greatly vary due to different features of 6‐CF and AnnCy3, as well as cell morphologies in control and treatment conditions. After subtraction steps, a Merge binary image was eroded back after using Erode operation,^7^ and particles in this image were analysed with the right parameter set for circularity as stated in Table 1. The total counts for AnnCy3, CF‐6 and Merge images were summarised and saved in excel sheet (Summary sheet) automatically (Figure 8).

### 3.4 Data processing

Using the information (Count) from Summary sheet, the following simple formula can be adopted to generate information about total number of cells:

$$\begin{array}{ccc} Total\;cells & = & 6-CF\;subt\;merge+AnnCy3\;subt\;Merge\\ & & +\;Merge. \end{array}$$

In addition to this information, total area, average size, %area, mean and IntDen (Integrated Density) values are also generated, providing supplementary details to the number of viable, apoptotic or necrotic cells. Together with Summary sheet, Deconvolved images (6‐CF, AnnCy3) and Mask images (6‐CF only overlay mask, AnnCy3 only overlay mask, Merge overlay mask) are also saved in the directory specified by the user providing a detailed insight about ApoNecV outcome to the user.

### 3.5 ApoNecV versus manual cell counting

For control and treated cell samples, two macros ApoNecV C (control) and ApoNecV TR (treated) were generated. To test the ApoNecV C/TR macros reliability, the identical set of images was analysed using both ApoNecV C/TR and manual cell counting methods performed by two different individuals to guarantee unbiased results. Below, Table 2 with the results gathered from both methods is presented.  Here, three negative control images and three treatment images were analysed. The agreement of the values from manual and ApoNecV C/TR measurements was assessed using ICCs (Intraclass Correlation Coefficients) with 95% CI (confidence interval). ICC = 0.993 for apoptotic measurements suggests excellent agreement between Manual versus ApoNecV C/TR approaches. Similar statistical evaluation was carried out for necrotic (ICC = 0.998) and viable (ICC = 1.000) results. The p‐value interpretation (*p* < 0.0001) indicates that the ICCs are highly statistically significant.

**TABLE 2:** A comparison of manual and ApoNecV C/TR image processing after staining HeLa cells with APOAC detection kit is presented. Numbers indicate counts for apoptotic, necrotic and viable cells. These numbers were then summed up to generate a field ‘Total’ representing total number of cells in the sample. The difference in values is listed in the column DIF.

∼	∼	Neg. control		DIF	ZnPc treatment		DIF
Img	Cells	Manual	ApoNecV C	∼	Manual	ApoNecV TR	∼
1	Apoptotic	5	5	0	59	50	9
∼	Necrotic	5	3	2	88	110	22
∼	Viable	308	316	8	0	8	8
∼	Total	318	324	6	147	168	21
2	Apoptotic	2	1	1	19	19	0
∼	Necrotic	1	1	0	65	65	0
∼	Viable	400	404	4	3	6	3
∼	Total	403	406	3	87	90	3
3	Apoptotic	11	9	2	51	51	0
∼	Necrotic	9	7	2	259	264	5
∼	Viable	425	424	1	15	15	0
∼	Total	445	440	5	325	330	5

## 4 DISCUSSION

The ApoNecV implementation in microscopy data analysis offers convenient image processing with outputs representing apoptotic, necrotic and viable cells in case of both untreated and treated conditions. When compared with a traditional manual cell counting method, this macro carried out nearly identical result values representing a prompt tool that can be easily used to facilitate the cell death analysis.

This ApoNecV macro was applied and validated to cell cultured cultivated in vitro, specifically treated and handled according to APOAC kit (Sigma‐Aldrich), which was designed for detection of apoptotic, necrotic and viable cells in a variety of cell samples. Thus, the ApoNecV should be applied for cell samples treated with APOAC cell death detection kit under the same conditions as officially recommended in Sigma‐Aldrich protocol.

**FIGURE 9:**
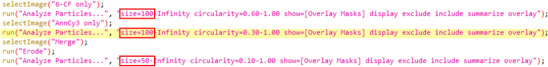
Size values for 6‐CF only, AnnCy3 only and Merge images that can be modified for more relevant results.

Importantly, Overlay masks provide the ApoNecV macro user with information about the fluorescence signal that was included in the analysis. This signal can be recognised as it appears coloured and numbered according to its position in the analysed image. Different cell lines can express distinct morphological features causing a potential imprecision in calculations. If there is a significant amount of signal that was not included in the analysis, a script can be easily adjusted to adopt these changes. The most convenient way is to change the value for size from ‘size = 100–infinity’ to ‘size = 50–infinity’ if more signal should be included for a particular image channel (6‐CF only, AnnCy3 only or Merge) in ApoNecV. Conversely, if less signal should be analysed, then a simple conversion to ‘size = 100–infinity’ can be implemented as shown in Figure 9.

Special attention should be given to images with lower signal‐to‐noise ratio. In general, it would be advisable to avoid images with a low signal‐to‐noise ratio by choosing the appropriate immersion medium. Additionally, background subtraction and deconvolution processes included in ApoNecV macro should be sufficient for noise suppression. However, if these actions do not have the expected effect on image quality, it is important to carefully assess the Overlay masks. Size can be easily adjusted as already described above to include/exclude signal from analysis. If changing the size value does not help significantly, a manual thresholding can be adopted instead of automated one described in section Mask Generation. Also, due to morphological differences between viable and treated cells, two types of macros were generated with a slightly different arrangement. Here, the ApoNecV C should be used for healthy or relatively healthy cells, while ApoNecV TR should be used for cells that are undergoing cell death. The use of one or the other type of macro depends on the user's assessment and should be done in accordance with cell damage, which can be easily recognised during cell imaging with a fluorescent microscope. If the red signal predominates in analysed images, the ApoNecV TR macro should be used. On the contrary, it is necessary to use ApoNecV C macro if the green signal prevails. To decide whether ApoNecV C or TR should be applied to images with comparable ratio of red and green fluorescence, the Overlay masks generated by each macro type can be used. If excessive background noise is observed in Overlay mask resulting from one of these macros, the other one should be applied to improve accuracy and reduce background interference.

Importantly, the ApoNecV macro was designed and tested with Annexin V‐Cy3^TM^ Apoptosis Detection Kit (APOAC, Sigma‐Aldrich), but can be also used with other dyes exhibiting the same fluorescent properties. For example, Calcein‐AM, which stains live cells green after diffusing across intact membranes and being cleaved by intracellular esterases, can be used effectively with Ethidium Homodimer‐1 (EthD‐1) staining nucleic acids after membrane compromise providing red fluorescence (PTG Lab, n.d.).^12^ Additionally, Annexin V‐FITC which emits green fluorescence can be used to detect early apoptosis and can be combined with other dyes such as propidium iodide (PI) with red fluorescence labelling necrotic cells. In this way, early apoptotic cells (stained only with Annexin V‐FITC) can be distinguished from late apoptotic/necrotic cells (stained with both Annexin V‐FITC and PI) (BioVision, n.d.).^13^ However, attention must be given to incorrect names in the final excel outcome, as the names are related to official probes from Annexin V‐Cy3^TM^ Apoptosis Detection Kit (APOAC, Sigma‐Aldrich). Similarly, a protocol can be adjusted with Zombie Dye Series (BioLegend, 2024)^14^ or Caspase‐3/7 Assay kit (Thermo Fisher Scientific, n.d.)^15^ according to protocol design.

## 5 CONCLUSION

In this study, we present an efficient automated image processing method designed for accurate cell counting and cell death classification. These data are generated using two macros that were created in FiJi Macro Language providing information about number of viable, apoptotic and/or necrotic cells in sample in much more simplified and convenient way when compared to traditional manual cell counting. In the first step, image postprocessing is performed, therefore generating deconvolved images with background subtraction enabling correct image analysis. Additionally, overlay masks are also generated to provide a user with exact information about the number of cells that were involved in analysis.  Evaluating ICCs, excellent agreement was found between Manual and ApoNecV C/TR approaches suggesting the relevancy of these macros. The ApoNecV C/TR macros can be used after staining cell samples with Annexin V‐Cy3^TM^ Apoptosis Detection Kit (APOAC, Sigma‐Aldrich) and can be found on GitHub platform (Kolarikova, 2024).^16^

## AUTHOR CONTRIBUTIONS

All authors contributed equally to this work. Specifically, Kolarikova M., Tesarik J. and Hosikova B. conceptualised and designed the study; Kolarikova M. and Hosikova B. performed the experiments; Tesarik J. and Langova K. analysed the data; and Hosikova B., Kolarova H., Tesarik J. and Kolarikova M. wrote the manuscript. All authors reviewed and approved the final manuscript.

## CONFLICT OF INTEREST STATEMENT

The authors declare no conflict of interest.

## CODE AVAILABILITY

The code used in this study is available on GitHub at (GitHub – kolarikma/Ap…). Users are encouraged to cite this code in any publications or presentations that utilise it. The citation for the code is as follows: Kolarikova M, Hosikova B, Tesarik J, Langova K, Kolarova H.*ApoNecV: A Macro for Cell Death Type Differentiation*, GitHub, 2024, (GitHub – kolarikma/Ap…). For detailed instructions on how to use the code, please refer to the README file in the repository.

## Data Availability

Research data is available from the corresponding author on request.
